# Coronary calcium score in COVID-19 survivors: Association with cardiac injury and cardiac function after 6 weeks

**DOI:** 10.1016/j.ahjo.2023.100280

**Published:** 2023-02-23

**Authors:** R.A. Groen, M.A. de Graaf, J.L. Stöger, P.R.M. van Dijkman, J.W. Jukema, M.J. Schalij, J.J.M. Geelhoed, M.L. Antoni

**Affiliations:** a Leiden University Medical Centre, Department of Cardiology, Albinusdreef 2, 2333ZA Leiden, the Netherlands; b Leiden University Medical Centre, Department of Radiology, Albinusdreef 2, 2333ZA Leiden, the Netherlands; c Leiden University Medical Centre, Department of Pulmonology, Albinusdreef 2, 2333ZA Leiden, the Netherlands; d The Netherlands Heart Institute, Moreelsepark 1, 3511 EP Utrecht, The Netherlands

**Keywords:** COVID-19, Out-patient clinic, Echocardiography, Coronary artery calcium

## Abstract

**Aims:**

Cardiac manifestations are common in COVID-19, often elevated serum troponin levels or myocardial dysfunction on trans-thoracic echocardiography (TTE) is observed. Both parameters are associated with increased in-hospital mortality. Possibly, subclinical coronary atherosclerosis plays a role, of which severity can be assessed by calculating the coronary artery calcium (CAC) score. This study aims to determine the relation between coronary atherosclerosis and cardiac manifestations in COVID-19 survivors.

**Methods:**

This study was conducted at the Leiden University Medical Center. All patients admitted for COVID-19 were included and scheduled for a 6-week follow-up visit with trans-thoracic echocardiography (TTE). CAC was assessed according to an ordinal score on non-gated, non-contrast enhanced computed tomography of the chest. Patients with and without CAC were compared on cardiac injury as reflected by elevated serum troponin levels and impaired cardiac function assessed through TTE.

**Results:**

In total, 146 patients were included. Mean age was 62 years and 62 % of the patients were male. During admission, patients with CAC showed significantly higher levels of troponin (19 ng/L vs 10 ng/L; *p* < 0.01). Overall, mild echocardiographic abnormalities were seen; 12 % showed reduced left ventricular function (left ventricular ejection fraction of <50 %) and 14 % reduced right ventricular function (tricuspid annular planar systolic excursion ≤17 mm). Following multivariable adjustments, there was no significant relation between CAC and myocardial function at 6 weeks.

**Conclusion:**

The present study shows that coronary atherosclerosis is associated with cardiac injury in COVID-19 survivors. However, no significant relation with impaired cardiac function was demonstrated.

## Highlights

In COVID-19 survivors:

• Coronary calcium is associated with significantly elevated serum troponin-T levels.
• Mild echocardiographic abnormalities are observed at 6 weeks post-discharge.
• Coronary calcifications are not significantly related to cardiac dysfunction on TTE.

## 1 Introduction

Starting summer 2020 studies reported cardiac manifestations, presented as decreased left ventricular ejection fraction (LVEF) and elevated serum troponin levels in 20 % of COVID-19 patients [1], [2], [3], [4], [5].

The SARS-CoV-2 virus induces a pro-inflammatory cytokine response that arguably plays a role in causing cardiac injury and decreased myocardial function [6]. The exact pathological mechanism is yet to be determined. Some studies show an association between cardiac manifestations and myocarditis, suggesting direct viral injury combined with immune-mediated secondary cardiac damage [6], [7], [8].

Another possible factor in COVID-19 related cardiac manifestations concerns pre-existent subclinical atherosclerosis [9], [10]. The pathophysiology could be either secondary cardiac damage due to a mismatch in myocardial oxygen supply and demand or coronary plaque destabilization [8]. Hypothetically, patients with coronary artery disease are more prone to cardiac complications due to COVID-19.

Patients' coronary atherosclerosis burden can be estimated by assessment of coronary artery calcium (CAC), demonstrated by previous studies as a valuable prognostic factor in determining COVID-19 patients' survival [11]. They identified CAC as a high-risk marker for patients' prognosis and as predictor for admission to an intensive care unit [12]. CAC can be readily assessed on routinely performed computed tomography (CT) of the chest as well [13]. Chest CT was performed in nearly all admitted COVID-19 patients for evaluation of pulmonary disease severity, simultaneously these scans could be employable in determining COVID-19 patients' coronary atherosclerosis burden. To investigate the relation between CAC and COVID-19 related cardiac manifestations, the present study compared COVID-19 survivors with and without CAC and their differences in cardiac injury assessed through troponin and cardiac function at 6 weeks follow-up.

## 2 Methods

### 2.1 Patients

The patient population consists of consecutive patients admitted with a SARS-CoV-2 infection at the Leiden University Medical Centre (LUMC) and scheduled for a clinical evaluation and trans-thoracic echocardiography (TTE) after six weeks post-discharge. During hospital admission Hs-troponin T levels of all patients were assessed as marker of cardiac injury, using a cut-off point of 14 ng/L. This population has been previously described [14]. In all admitted patients a chest CT was performed.

For this retrospective analysis, we included COVID-19 patients who underwent a non-gated chest CT without intravenous contrast administration. All patients with known CAD, percutaneous coronary intervention or coronary artery bypass graft were excluded. The hospital's ethical review board approved the study. All patients admitted to the hospital were given a letter stating that their data could be used for research purposes, and that they could opt out upon request. None of the patients have declined consent.

#### 2.1.1 Trans-thoracic echocardiography

TTE was performed 6 weeks post-discharge, using standard systems (General Electronics Healthcare, Vivid E95, Horten, Norway). The two-dimensional and Doppler data were evaluated using EchoPac. For this study, all exams were evaluated by an experienced observer blinded from all other relevant clinical data.

Analysis of left ventricular (LV) and right ventricular (RV) function was performed [14]. LV function was calculated using the Simpson biplane method and divided in four groups (normal of >52 % for males and >54 % for females and three abnormal subgroups of 40–52/54 %, 30–40 % and <30 % for both male and female patients). LV global longitudinal strain (GLS) values were measured using speckle-tracking in apical four, two and three chamber views and defined as normal or abnormal (≤−16 % vs >−16 %). For RV function the tricuspid annular systolic planar excursion (TAPSE) was measured, defined as normal and abnormal (>17 mm vs ≤17 mm). The tricuspid annular peak systolic velocity (S′) was calculated by tissue doppler imaging. RV fractional area change, RV-strain and RV end-diastolic diameter (RVEDD) were measured in four-chamber view. S′ was defined as normal vs abnormal (>10 cm/s vs ≤10 cm/s) and RV FAC (>35 % vs ≤35 %). For RV-strain −23 % was used as cut-off value [15], [16]. Diastolic dysfunction was graded on a semiquantitative scale (grade 0–3) using an integrated method incorporating E/A ratio, E′, E/E′, left atrial volume index (LAVI) and tricuspid regurgitation gradient according to current guidelines [17], [18].

### 2.2 Image acquisition and evaluation

Non-gated, non-enhanced chest CT was performed upon hospital admission as part of routine care (Canon Medical Systems, The Netherlands). CAC was visually assessed using a previously described ordinal score [19]. All data was acquired by one observer without access to other baseline variables or echocardiography data. The rationale for using this ordinal score was the technical limitations of applying Agatston score on non-gated chest CT and the previously described strong correlation between both [13], [20]. Calcification of the right coronary artery, the left main, the left anterior descending and the ramus circumflex were assigned a score ranging from 0 to 3. Score 0 indicated no calcification, whereas 1 indicated less than a third calcified, 2 less than two third and 3 more than two third calcified. The summed score ranged from 0 to 12. This technique correlates with prognosis of patients with CAD and patients with COVID-19 [11], [12], [13]. For the first analysis patients were divided into two groups; the ‘no calcium’ group defined as a score of 0 versus the ‘calcium>0’ group. A complementary analysis was performed, incorporating CAC-severity. Patients were divided into 3 categories, defined as no (0), mild (1–3) and severe (4–12) [21], [22].

### 2.3 Statistical analysis

Statistical analyses were performed using IBM SPSS version 25.0. Continuous variables were reported as mean ± standard deviation, dichotomous variables as number (%). Calcium-groups were compared using an independent sample *t*-test or One-way ANOVA for numerical outcomes and a Chi-square test for dichotomous outcomes. Finally, multivariate logistical regression models to adjust for the two most important confounders (i.e. age and gender) were created. To avoid over fitting of the model, only a small selection of the univariate significant variables was entered into the multivariate model (i.e., age, male gender and CAC-risk category). Our primary outcome parameters were troponin T, LVEF, LV GLS, RV-strain, TAPSE, S′ and diastolic dysfunction. A two side p-value <0.05 was considered significant.

## 3 Results

**Fig. 1:**
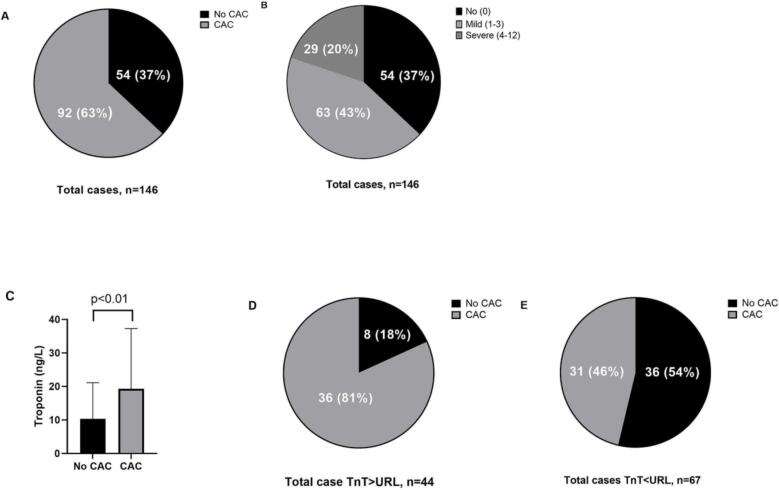
Distribution of CAC and troponin among patients. Distribution of CAC and troponin among patient with and without CAC. A: shows the distribution of patients with and without coronary calcium, n (%). B: shows the distribution of CAC-severity among patients, n (%). C: shows troponin levels during admission. D: shows distribution CAC among patients with elevated troponin levels >URL (14 ng/L), n (%). E: shows distribution of CAC among patients with normal troponin levels, n (%).

The baseline characteristics are described in Table 1. The mean age was 62 years (SD 12.3) and 62 % were male, both variables were significantly different between the patients with and without CAC. Overall 54 patients showed no CAC on their non-gated chest CT. Most patients (*n* = 63, 43 %) showed mild CAC on non-gated chest CT and 20 % (*n* = 29) of patients showed severe CAC on non-gated chest CT. The CAC > 0-group (i.e. CAC mild and CAC severe) showed a significant higher percentage of hypercholesterolemia (34 % vs 11 %; *p* < 0.01), hypertension (44 % vs 24 %; *p* = 0.02) and chronic kidney disease (CKD) (13 % vs 2 %; p = 0.02). They had significant higher serum levels of troponin T during admission (19 ng/L vs 10 ng/L; *p* < 0.01) and significantly more troponin T levels above the upper reference limit (*p* < 0.01). Fig. 1 shows the distribution of troponin and CAC among the patients. Between the CAC-severity groups troponin levels were significantly different (*p* < 0.01). The severe CAC-group showed a significantly higher percentage of patients with troponin levels >URL (18 % vs 51 % vs 63 %; p < 0.01), as described in Table 2. There was no difference in number of patients admitted to the ICU.

**Table 1:** Baseline characteristics of the study population^a^, *n* = 146.

	All	No CAC (n = 54)	CAC > 0 (n = 92)	p-Value
Age, years	61.9 ± 12.3	53.9 ± 10.7	66.6 ± 10.6	**<0.01**
Sex, men, %	91 (62.3)	27 (50.0)	64 (69.6)	**0.02**
BMI, kg/m^2^	28.6 ± 5.5	29.3 ± 5.9	28.2 ± 5.2	0.23

History of				
Hypertension, %	53 (36.3)	13 (24.1)	40 (43.5)	**0.02**
Diabetes, %	35 (24.0)	12 (22.2)	23 (25.0)	0.70
Hypercholesterolemia, %	37(25.3)	6 (11.1)	31 (33.7)	**<0.01**
Cardiovascular disease, %				
AF/AFI	10 (6.8)	1 (1.9)	9 (9.8)	0.07
Valvular abn	5 (3.4)	0 (0.0)	5 (5.4)	0.08
CVA/TIA	7 (4.8)	1 (1.9)	6 (6.5)	0.20
PVD	4 (2.7)	0 (0.0)	4 (4.3)	0.12
HFrEF	1 (0.7)	0 (0.0)	1 (1.1)	0.44
HFmEF	0 (0.0)	0 (0.0)	0 (0.0)	
HFpEF	0 (0.0)	0 (0.0)	0 (0.0)	
Smoking, %	21 (14.4)	8 (14.8)	13 (14.1)	0.90
CKD, %	13 (8.9)	1 (1.9)	12 (13.0)	**0.02**

In hospital				
Time in hospital (days)	16.5 ± 14.9	20.0 ± 19.6	14.3 ± 10.8	0.12
CRP maximum, mg/L	146.5 ± 125.0	156.6 ± 142.9	140.9 ± 114.5	0.47
Troponin T max, ng/L	15.7 ± 16.1	10.3 ± 10.8	19.3 ± 18.0	**<0.01**
Troponin T > URL, %	44 (30.1)	8 (18.2)	36 (81.8)	**<0.01**
Troponin *T* > 3 URL^b^ ng/L, %	8 (7.2)	1 (2.3)	7 (10.4)	0.10
Pulmonary embolism, %	21(14.4)	9 (16.7)	12 (13.0)	0.55
ICU admission	34 (23)	17 (31.5)	17 (18.5)	0.07
Time ICU (days)	8.0 ± 12.1	11.7 ± 15.9	5.6 ± 8.1	0.38
Kidney function				
eGFR, mL/min/1.73m2	71.9 ± 20.2	76.0 ± 16.1	69.6 ± 21.9	0.07
Creat max, μmol/L	98.8 ± 63.2	87.9 ± 27.6	105.2 ± 76.2	0.11
Urea max, mmol/L	10.2 ± 8.1	9.4 ± 9.1	10.4 ± 7.4	0.61

BMI = Body mass index, AF = Atrial fibrillation, AFI = Atrial flutter, Abn = Abnormalities, CVA/TIA = Cerebrovascular accident/Transient ischemic attack, PVD = Peripheral vascular disease, HFpEF, HFmEF, HFrEF = Heart failure with preserved, mid-range, reduced ejection fraction, CKD = Chronic kidney disease, CRP = C-reactive protein, URL = Upper reference limit, ICU = Intensive care unit, eGFR = Estimated glomerular filtration rate, Creat = Creatinine.

a All data are presented as mean ± SD or as number (%). P-values <0.05 are written in **bold**.

b URL = 14 ng/L.

**Table 2:** Troponin and primary TTE outcomes for CAC categories^a^, n = 146.

	All	No CAC (n = 54)	CAC mild (n = 63)	CAC severe (n = 29)	p-Value
Troponin T max, ng/L	15.7 ± 16.1	10.3 ± 10.8	17.9 ± 15.4	23.6 ± 24.6	**<0.01**
Troponin T > URL, %	44 (30.1)	8 (18.2)	26 (51.0)	10 (62.5)	**<0.01**
LVEF, %	55.8 ± 8.1	55.8 ± 6.9	57.5 ± 6.5	51.7 ± 11.7	**<0.01**
>52 (M), >54 (F)	97 (66.9)	36 (66.7)	49 (77.8)	12 (42.9)	0.36
40–52 (M)/54 (F)	45 (31.0)	17 (31.4)	13 (20.6)	15 (53.5)	
30–40	1 (0.7)	0 (0.0)	1 (1.6)	0 (0.0)	
<30	2 (1.4)	1 (1.9)	0 (0.0)	1 (3.6)	
LV GLS, %	−16.7 ± 2.8	−17.0 ± 2.9	−17.1 ± 2.7	−15.1 ± 2.3	**0.01**
≤−16	79 (65.8)	32 (71.1)	38 (69.1)	9 (45.0)	0.10
>−16	41 (34.2)	13 (28.9)	17 (30.9)	11 (55.0)	
TAPSE, mm	20.95 ± 3.2	20.9 ± 3.1	21.5 ± 3.2	19.8 ± 3.4	0.06
≤17	20 (13.7)	6 (11.1)	6 (9.5)	8 (27.6)	0.05
>17	126 (86.3)	48 (88.9)	57 (90.5)	21 (72.4)	
S′, cm/s	13.4 ± 3.34	13.4 ± 3.0	13.8 ± 3.4	13.0 ± 2.9	0.56
≤10	21 (15.0)	7 (13.5)	8 (13.3)	6 (21.4)	0.57
>10	119 (85.0)	45 (86.5)	52 (86.7)	22 (78.6)	
RV strain, %	−23.1 ± 5.8	−22.4 ± 6.0	−24.2 ± 5.0	−22.6 ± 6.9	0.25
≤−23	74 (56.1)	24 (48.0)	36 (64.3)	14 (53.8)	0.23
>−23	58 (43.9)	26 (52.0)	20 (35.7)	12 (46.2)	
Diastolic dysfunction grade, %					**0.03**
0	86 (58.9)	40 (74.1)	32 (50.8)	14 (48.3)	
I	56 (38.4)	13 (24.1)	30 (47.6)	13 (44.8)	
II	4 (2.7)	1 (1.9)	1 (1.6)	2 (6.9)	
III	0 (0.0)	0 (0.0)	0 (0.0)	0 (0.0)	

LVEF = Left ventricular ejection fraction, LV GLS = Left ventricular global longitudinal strain, TAPSE = Tricuspid annular planar systolic excursion, S′ = Tricuspid annular systolic peak velocity, URL = Upper reference limit.

a All data are presented as mean ± SD or as number (%). P-values <0.05 are written in **bold**.

**Fig. 2:**
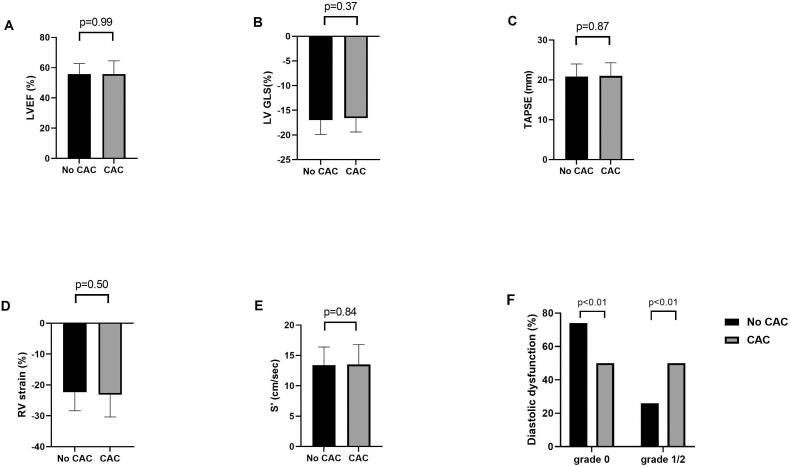
Primary outcome parameters on TTE. LV, RV and diastolic parameters compared between patients with and without CAC. A: shows left ventricular ejection fraction. B: shows left ventricular global longitudinal strain. C: shows tricuspid annular planar systolic excursion. D: shows right ventricular strain. E: shows tricuspid annular systolic peak velocity. F: shows diastolic dysfunction grade 0 and grades 1 & 2.

Table 2, Table 3 show the TTE parameters measured at 6 weeks post-discharge. Fig. 2 shows LV, RV and diastolic parameters compared between patient with and without CAC.

**Table 3:** Echocardiography at 6 weeks post discharge^a^, n = 146.

	All	No CAC (n = 54)	CAC > 0 (n = 92)	p-Value
LVESV, mL	40.6 ± 19.3	41.5 ± 18.9	40.6 ± 19.2	0.79
LVEDV, mL	90.6 ± 34.2	92.3 ± 29.6	90.7 ± 35.6	0.79
LVEF, %	55.8 ± 8.1	55.8 ± 6.9	55.8 ± 8.8	0.99
>52 (M), >54 (F)	97 (66.9)	36 (66.7)	61 (67.0)	0.55
40–52 (M)/54 (F)	45 (31.0)	17 (31.4)	28 (30.8)	
30–40	1 (0.7)	0 (0.0)	1 (1.1)	
<30	2 (1.4)	1 (1.9)	1 (1.1)	
LV GLS, %	−16.7 ± 2.8	−17.0 ± 2.9	−16.6 ± 2.8	0.37
≤−16	79 (65.8)	32 (71.1)	47 (62.7)	0.35
>−16	41 (34.2)	13 (28.9)	28 (37.4)	
RVEDD, mm	33.9 ± 6.2	33.1 ± 6.9	34.7 ± 4.6	0.08
TAPSE, mm	20.95 ± 3.2	20.9 ± 3.1	21.0 ± 3.3	0.87
≤17	20 (13.7)	6 (11.1)	14 (15.2)	0.49
>17	126 (86.3)	48 (88.9)	78 (84.8)	
S′, cm/s	13.4 ± 3.34	13.4 ± 3.0	13.5 ± 3.25	0.84
≤10	21 (15.0)	7 (13.5)	14 (15.9)	0.70
>10	119 (85.0)	45 (86.5)	74 (84.1)	
RV FAC, %	42.6 ± 5.8	43.0 ± 11.1	42.4 ± 7.9	0.69
≤35	26 (17.9)	11 (20.8)	15 (16.3)	0.50
>35	119 (82.1)	42 (79.2)	77(83.7)	
RV strain, %	−23.1 ± 5.8	−22.4 ± 6.0	−23.7 ± 5.7	0.50
≤−23	74 (56.1)	24 (48.0)	50 (61.0)	0.15
>−23	58 (43.9)	26 (52.0)	32 (39.0)	
Diastolic dysfunction grade, %				**0.02**
0	86 (58.9)	40 (74.1)	46 (50.0)	
I	56 (38.4)	13 (24.1)	43 (46.7)	
II	4 (2.7)	1 (1.9)	3 (3.3)	
III	0 (0.0)	0 (0.0)	0 (0.0)	
E/A	1.0 ± 0.5	1.0 ± 0.3	1.0 ± 0.6	1.00
E′ average, cm/s	8.8 ± 3.2	8.9 ± 3.0	8.9 ± 3.2	0.90
E/E′	7.7 ± 3.3	7.4 ± 2.6	7.9 ± 3.5	0.32
LAVI, mL/m^2^	28.5 ± 11.0	27.0 ± 8.1	29.7 ± 12.0	0.15
TI gradient, %				**0.04**
≤34 mmHg	110 (75.9)	46 (85.2)	64 (70.3)	
>34 mmHg	35 (4.9)	8 (14.8)	27 (29.7)	

LVESV = left ventricular end systolic volume, LVEDV = Left ventricular end diastolic volume, LVEF = Left ventricular ejection fraction, LV GLS = Left ventricular global longitudinal strain, RVEDD = Right ventricular end diastolic diameter, TAPSE = Tricuspid annular planar systolic excursion, S′ = Tricuspid annular systolic peak velocity, RV FAC = Right ventricular fractional area change, E = Early ventricular filling velocity, A = Late ventricular filling velocity, E' = Diastolic mitral annular velocity, LAVI = Left atrial volume index, TI = Tricuspid insufficiency.

a All data are presented as mean ± SD or as number (%). P-values <0.05 are written in **bold**.

### 3.1 Left ventricular function

Two third (67 %) of patients showed a normal LVEF. LVEF of 40–52/54 % was present in 31 % of patients, <1 % had LVEF 30–40 % and 1 % LVEF <30 %. The CAC-severity groups had similar mean LVEF, all normal (>50 %). Within the mild CAC-group 78 % of patients showed normal LVEF versus only 43 % in the severe CAC-group. LVEF of 40–52/54 was respectively 20 % vs 53 %. In 34 % of all patients LV GLS was abnormal (>−16 %), in patients with a severe CAC-score the majority showed an abnormal LV GLS. No significant difference between patients with and without CAC (29 % vs 37 %; *p* = 0.35).

### 3.2 Right ventricular function

The RV dimension and function assessed by RVEDD, TAPSE, S′, RV FAC and RV strain did not differ significantly between patients with and without CAC. No significant difference between the CAC-severity groups regarding abnormmal RV strain (26 % vs 20 vs 12; *p* = 0.23) was observed. TAPSE and S′ were similar for all three categories.

### 3.3 Diastolic dysfunction

Of all patients, 59 % showed normal diastolic function. 38 % showed grade I dysfunction, 3 % showed grade II and none of the patients showed grade III. Within the CAC > 0-group abnormal diastolic function occurred more frequently than in the no CAC-group (50 % vs 74 %; *p* = 0.02), more patients showed grade I and II dysfunction. Diastolic dysfunction was significantly different between the CAC-severity groups (49 % vs 52 %; *p* = 0.03).

### 3.4 Logistic regression

The regression models are shown in Table 4. Both univariate regressions for CAC, age and gender, and a multivariate regression adjusted for age and gender were made. In univariate regression CAC and elevated serum troponin T levels correlated significantly, though not in multivariate analysis. All parameters for LV and RV dysfunction showed no significant relation to CAC in univariate and multivariate regression. Diastolic dysfunction was significantly correlated to CAC > 0 in univariate but not in multivariate analysis. Additionally, the CAC categories correlated significantly with both LV GLS and diastolic dysfunction in univariate regression, though neither in multivariate regression.

**Table 4:** Logistical regression for CAC-score, age and gender⁎, n = 146.

	Troponin T, ≤14 vs >14 ng/L			LVEF, ≤50 vs >50 %			LV GLS, ≤−16 vs >−16 %			TAPSE, ≤17 vs >17 mm			S’, ≤10 vs >10 cm/s			RV strain, ≤−23 vs >−23 %			Diastolic dysfunction grade, 0 vs I/II		
**Univariate**																					
CAC>0	5.23	2.12;12.91	**<0.01**	0.44	0.14;1.41	0.17	1.47	0.67;3.25	0.35	0.70	0.25;1.94	0.49	0.82	0.31;2.19	0.70	0.59	0.29;1.20	0.15	2.86	2.37;5.95	**<0.01**
CAC-score	3.03	1.63;5.65	**<0.01**	0.48	0.24;0.95	**0.04**	1.63	0.95;2.80	0.07	0.56	0.29;1.07	0.08	0.77	0.41;1.43	0.41	0.82	0.51;1.31	0.41	1.83	1.15;2.91	**0.01**
Age	1.08	1.04;1.13	**<0.01**	0.98	0.94;1.02	0.23	1.02	0.99;1.05	0.27	0.98	0.94;1.02	0.28	0.96	0.92;1.00	0.06	1.01	0.98;1.04	0.46	1.09	1.05;1.13	**<0.01**
Gender	1.74	0.74;3.85	0.17	0.59	0.20;1.76	0.35	1.18	0.54;2.60	0.68	0.67	0.24;1.87	0.45	2.02	0.79;5.14	0.14	0.52	0.25;1.07	0.07	0.95	0.48;1.88	0.89

**Multivariate**																					
CAC>0	2.58	0.95;7.02	0.07	0.58	0.15;2.18	0.42	1.21	0.47;3.06	0.70	0.97	0.29;3.22	0.96	1.23	0.38;3.97	0.74	0.43	0.18;1.04	0.06	1.20	0.51;2.92	0.67
CACscore	1.70	0.82;3.53	0.15	0.51	0.21;1.22	0.13	1.59	0.81;3.12	0.18	0.60	0.26;1.37	0.22	0.92	0.40;2.08	0.83	0.72	0.39;1.33	0.30	0.87	0.47;1.61	0.65

⁎ All data are presented as odds ratio, confidence interval, p-value. CAC = Coronary calcium, LVEF = Left ventricular ejection fraction, LV GLS = Left ventricular global longitudinal strain, TAPSE = Tricuspid annular systolic planar excursion, S’ = Tricuspid annular systolic peak velocity, RV = Right ventricle.

**Fig. 3:**
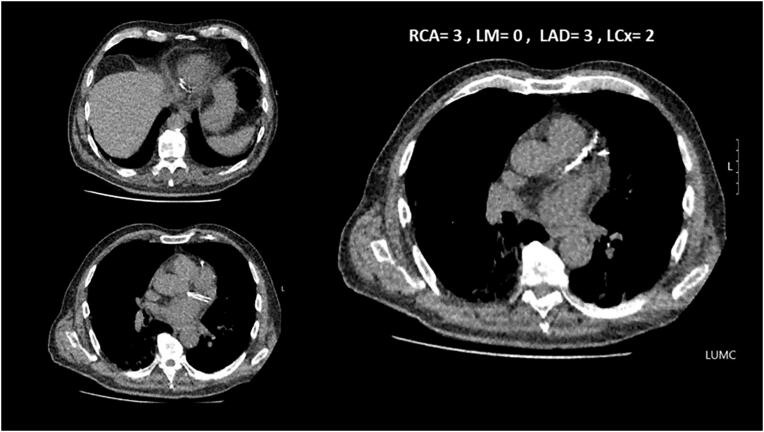
Case example of coronary calcium on chest CT. Example of a patient of 80 years old with a total calcium score of 8, classified as severe [11]. The calcified coronary arteries are the right, the left anterior descending and the circumflex. His medical history reports no previous cardiac problems. During his admission troponin T levels of 17 ng/L were measured, suggesting cardiac injury. Subsequently, echocardiography at 6 weeks post-discharge showed a LVEF of 62%, a LV GLS of −18.7 and a RV strain of −30.0, a TAPSE of 21.9 and S′ of 13. All considered normal [9]. This case represents an example of a patient with clear atherosclerosis and cardiac injury during admission but normal TTE values after 6 weeks.

Fig. 3 demonstrates a case example of a patient with coronary calcifications, elevated troponin levels during admission and normal TTE parameters during follow-up.

## 4 Discussion

This study compared COVID-19 survivors with and without CAC in relation to both cardiac function and injury, assessed by troponin release. Furthermore, a sub analysis of CAC-severity in relation to troponin release and cardiac function was performed. Significantly more patients in the CAC > 0-group showed elevated levels of troponin, especially patients in the severe CAC category. The association between CAC and elevated troponin showed a trend toward significance when corrected for age and gender.

For cardiac function, the primary outcome parameters LVEF, LV GLS, RV-strain, TAPSE, S′ and diastolic dysfunction showed small subclinical changes in cardiac function. No significant differences between patients with and without CAC were observed, except for diastolic dysfunction and abnormal LV GLS. Both correlated significantly with CAC in univariate regression, though not in multivariate regression. This can be explained by the relation between age and diastolic function and LV GLS dysfunction. [23], [24]

Studies have reported cardiac manifestations in a substantial percentage of COVID-19 patients. For instance, a study on the association of cardiac injury and mortality [1], described cardiac injury as a common complication. During admission, almost 20 % of 416 patients presented with significantly elevated troponin. In our study this was 30 %, which difference can be explained by a higher mean age of our population and potentially a higher atherosclerosis burden. Recently, a study on long-term cardiovascular outcomes of COVID-19 patients reported an increased cardiac risk, including cerebrovascular diseases, arrhythmias, heart failure, ischemic and non-ischemic heart disease, pericarditis, myocarditis, and thromboembolic disease [25]. Possibly, these patients had pre-existent subclinical cardiovascular disease which became manifest during admission for SARS-CoV2. Another study on coronary atherosclerosis in COVID-19 patients reported that CAC was not an independent predictor for in hospital mortality, but helped identify a high-risk population. [9] Complementary another study showed that previous CVD or increased biomarkers like troponin was part of a vulnerable phenotype [26], [27]. Alike our patients with (severe) CAC, who presented with elevated troponin levels.

Regarding cardiac dysfunction, a study with in-hospital performed echocardiography reported two-thirds of COVID-19 patients with elevated troponin levels, of whom 63 % showed reduced cardiac function. Myocardial injury, defined as any elevation in cardiac troponin at the time of clinical presentation or during the hospitalization, was associated with increased in-hospital mortality, especially when combined with echocardiographic abnormalities [28]. Another study (*n* = 1216) reported abnormal echocardiography in 50 % of patients [29]. Our study found a lower incidence of myocardial dysfunction. This can be explained by the timing in performing TTE (in-hospital vs. out-patient clinic). Two studies discussed the mechanism of RV and LV dysfunction in COVID-19 survivors [30], [31]. RV dysfunction was mostly presented as RV dilation with impaired S′ and RV FAC as a result of increased RV afterload due to an increase in pulmonary vascular resistance. LV dysfunction was presented as a decreased stroke volume combined with a smaller LV. Significantly impaired LV GLS was observed [30]. Comparing patients with and without myocardial injury defined by elevated hsTNT levels above the 99th percentile URL (0.014 ng/mL), the prior showed significantly enlarged RV, with RV FAC significantly impaired (*p* = 0.019). Regarding LV dysfunction, their LV GLS was significantly impaired (−13.9 %, *p* = 0.005) [31]. In these studies, echocardiography was performed during hospital admission. Our study performed TTE during follow-up and limited cardiac abnormalities were observed.

Still, some studies showed impaired cardiac function during follow-up. Ozer et al. reported impaired LV GLS in 38 % using a cut-off value of >−18 % [32]. This relatively low cut-off value could have resulted in overestimation of the incidence of abnormal LV strain. Akkaya et al. reported subclinical RV dysfunction in patients with mild severity of COVID-19, where significant difference in TAPSE was observed during follow-up between the control-group and the COVID-19 patients (resp. 24 mm ± 4.4 vs 22.4 mm ± 2.6) [33]. However, the observed differences were small and of limited clinical significance.

Van den Heuvel et al. assessed COVID-19 patients during both hospital admission and follow-up and demonstrated that the percentage of patients showing abnormal TTE during admission was smaller during follow-up (37 % vs 17 %). A trend toward normalization of cardiac function was observed [34]. Our study complements that trend by showing few abnormalities during follow-up. This improvement of myocardial function post-discharge underlines the hypothesis that demands ischemia causes cardiac manifestations in COVID-19.

To elaborate, studies on COVID-19 often report cardiac injury as high levels of cardiac markers [1], [2], [6], [7], [8], [11], [14] during admission. A review on COVID-19-related myocarditis explained these levels as a possible result of an increased oxygen demand during sepsis and an impaired supply due to CAD resulting in demand ischemia (Type 2 ischemia) [8]. This aggravated mismatch can be restored upon recovery. Our study supports this theory, with CAC-patients showing significantly elevated troponin T (i.e. cardiac injury) levels during admission and normal cardiac function during follow-up. Similarly, it has been noted in non-COVID-19 sepsis patients that the occurrence of myocardial injury (i.e. elevated troponin levels) was associated with cardiovascular morbidity [35].

Of interest, hypertension, hyperlipidemia and CKD have previously been associated with the degree of COVID-19 severity [36]. Moreover, CKD is related to coronary calcification and myocardial function could influence kidney function. This could have introduced bias since these factors all also related to CAD. Potentially, these risk factors are manifestations of the same pathological pathway and risk profile that causes severe COVID-19 disease.

### 4.1 Limitations

A limitation of this study is the relatively small sample size, due to the exclusion of patients with known cardiovascular history. In our study this was 12 % of patients vs 44 % in a previous publication [1], this is higher than our study due to their inclusion of deceased patients, while ours only includes survivors. The relatively small sample number of patients per category based on CAC-severity makes the results of the sub analysis less reliable.

A further limitation could be the low percentage of LV and RV dysfunction in this study population. Only mild abnormalities were observed during follow-up [14], which combined with a small sample size, could indicate that our study was underpowered to find an association between CAC and impaired cardiac function. Furthermore, no comparison was made between cardiac function on TTE at baseline and follow-up. Our analysis further lacks the inclusion of CMR imaging which could provide insight in myocardial injury and inflammation.

These limitations leave a ‘research gap’ in determining the pathophysiology and connection of CAD to COVID-19-related cardiac dysfunction. This could be studied more specifically with a larger cohort and with multiple-timepoint assessments of cardiac function during infection and recovery. Further investigation is warranted particularly in COVID-19 patients who exhibit troponin release during admission.

### 4.2 Conclusion

In conclusion this study shows an association of coronary atherosclerosis and cardiac injury in COVID-19 survivors and no significant association between coronary atherosclerosis and impaired cardiac function.

## Funding

The department of Cardiology received research grants from Biotronik, Medtronic and Boston Scientific. This funding was not applied to the current research.

## Declaration of competing interest

There is no conflict of interest for the present manuscript.
